# Kidney Transplant Complicated by Severe Parathyroid Hormone–Independent Hypercalcemia From Tumoral Calcinosis

**DOI:** 10.1016/j.xkme.2026.101360

**Published:** 2026-04-10

**Authors:** Abishek Volety, Nicholas Shaffer, Liam Biard, Timothy N. Holbrook, Simran Demla, Angelito F. Yango

**Affiliations:** 1 Nephrology Division, Department of Medicine, University of Maryland Medical Center, Baltimore, MD; 2 Internal Medicine, Baylor Scott & White All Saints Medical Center/Texas Christian University Medical School Graduate Medical Education, Fort Worth, TX; 3 Clinical and Transplant Nephrology, Baylor Scott & White All Saints Medical Center, Fort Worth, TX; 4 Clinical and Transplant Nephrology, Dallas Nephrology Associates, Fort Worth, TX

**Keywords:** Tumoral calcinosis, renal transplantation, hypercalcemia, hyperparathyroidism, dialysis

## Abstract

Tumoral calcinosis (TC) is a rare complication of progressive kidney failure, characterized by massive depositions of calcium and phosphate into the periarticular soft tissue. Although generally associated with hyperparathyroidism and an elevated calcium-phosphorus product, there are few curative treatments for this condition. Although kidney transplantation typically leads to complete resolution of TC, the early posttransplant course may paradoxically mobilize soft tissue calcium into the general circulation, causing severe hypercalcemia and renal allograft injury. We report a rare case of acute allograft injury from severe hypercalcemia in a kidney transplant recipient, with worsening of her preexisting TC. The hypercalcemia was resistant to standard and off-label therapies, including saline, loop diuresis, calcimimetics, and bisphosphonates. Eventually, this was resolved with the initiation of temporary hemodialysis and saline infusion. This patient’s severe pretransplant hyperparathyroidism, use of calcium-containing phosphate binders with activated vitamin D analogs, and prolonged dialysis dependence contributed to her unique clinical course. This case emphasizes the importance of individualized bone-mineral metabolism optimization in high-risk patients considered for kidney transplantation to prevent dangerous mineral shifts and preserve future allograft function.

## Introduction

Tumoral calcinosis (TC) is a rare clinical histopathologic syndrome presenting with painful calcified masses in the periarticular soft tissue regions.^1^ TC is typically seen in the setting of chronic kidney disease and is usually associated with hyperparathyroidism, hypercalcemia, and hyperphosphatemia.^1^^,^^2^ The standard treatment of this illness emphasizes lowering the calcium-phosphorus product via dietary restrictions and phosphate binders. Off-label therapies include calcimimetics, hemodialysis using a low-calcium dialysate, and surgical parathyroidectomy, which may be indicated in resistant cases.^1^, ^2^, ^3^, ^4^, ^5^ The current literature suggests kidney transplantation is the measure that typically leads to partial or complete resolution of TC. However, owing to the extended duration of time needed for resolution, complications are known to occur in the early posttransplantation course. Hypercalcemia from soft tissue release may cause allograft injury owing to vasoconstriction, volume depletion, and direct tubular damage.^2^

This report describes a case of acute allograft injury from severe hypercalcemia after kidney transplantation in a patient with TC, which was resistant to standard and off-label drug therapy. Severe pretransplant hyperparathyroidism and established TC may predispose to early posttransplant parathyroid hormone (PTH)-independent hypercalcemia and allograft injury. In this patient, timely initiation of low-calcium hemodialysis followed by saline infusion resulted in sustained recovery of calcium levels and allograft function.

## Case Report

A 50-year-old African American woman with dialysis-dependent chronic kidney disease secondary to HIV (human immunodeficiency virus)-associated nephropathy received a living unrelated kidney transplant after 5 years of peritoneal dialysis. Ten months before her transplant, she developed multiple palpable painful masses in her hips and right supraclavicular area. A diagnosis of TC was made based on radiography showing lobulated calcified masses in the soft tissue and periarticular regions of her pelvis and right shoulder.

Bone-mineral laboratory results in the 4 months before the transplant showed persistently elevated levels of phosphate (7-9 mg/dL), calcium (10-11 mg/dL), and intact PTH (iPTH) (800-1,100 pg/mL). Her peritoneal dialysis prescription consisted of 4 nocturnal exchanges (8 L total), using a 1.5% dextrose solution with 2.5 mEq/L calcium. She became oliguric in the final 15 months before her transplant. Her pretransplant dialysis Kt/V was 1.7, and her calcium-phosphorus product ranged from 70-99 mg^2^/dL^2^. Her medications included calcitriol, sevelamer, and cinacalcet.

The patient underwent a living unrelated kidney transplant with an unremarkable postoperative course. She received antithymocyte globulin induction with maintenance immunosuppression consisting of tacrolimus, mycophenolate mofetil, and prednisone. At discharge, her serum creatinine (Scr) and calcium levels were 1.4 and 9.4 mg/dL, respectively. At 2 weeks, Scr increased to 1.8 mg/dL, calcium to 12 mg/dL, with iPTH 77 pg/mL. She was instructed to restart cinacalcet (60 mg) and increase her fluid intake.

**Figure 1:**
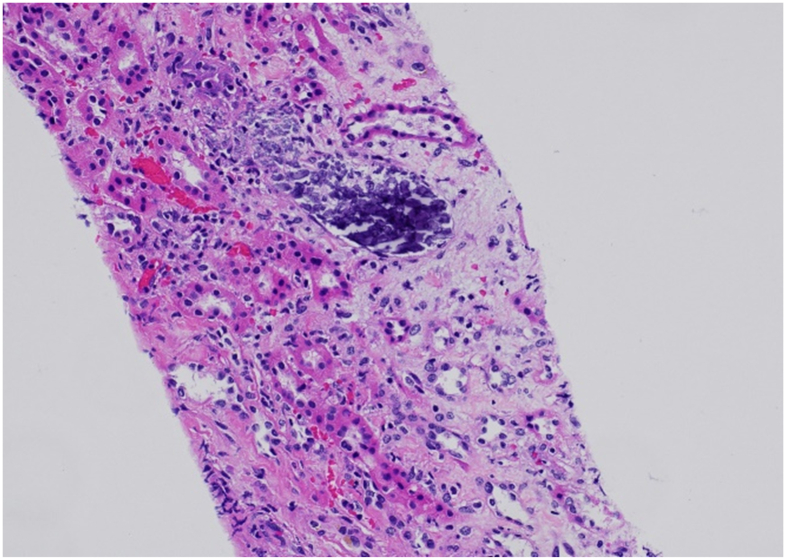
Transplanted kidney biopsy demonstrating peritubular calcium deposition.

**Figure 2:**
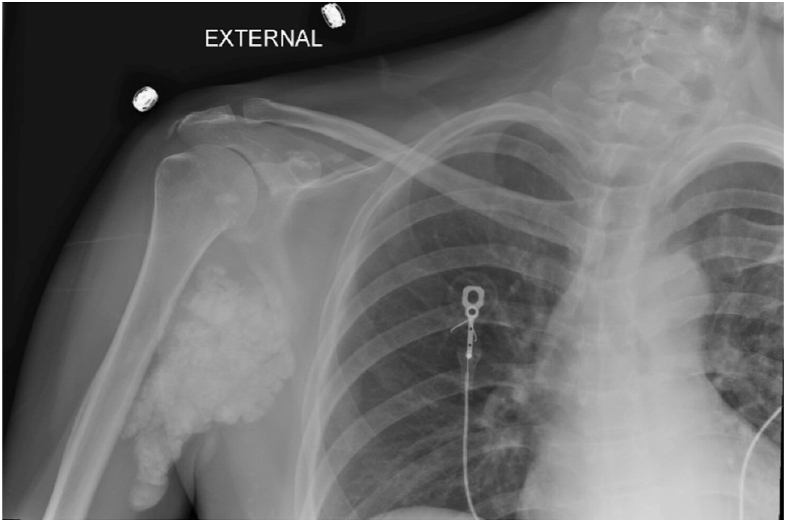
Initial right shoulder radiograph demonstrating a lobulated, calcified mass in the soft tissues around the right shoulder joint, measuring 115 x 59 mm.

**Figure 3:**
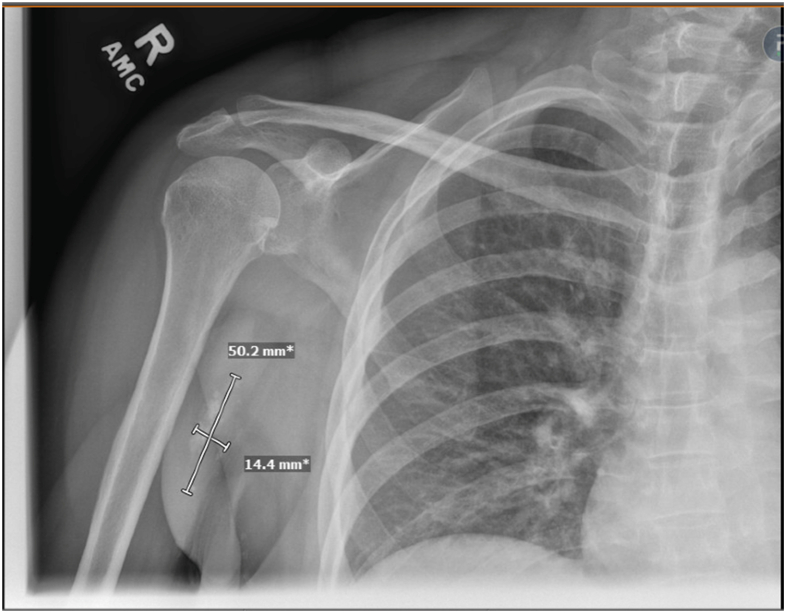
Follow-up radiograph of the right shoulder demonstrating near resolution of periarticular soft tissue calcium deposition (50 × 14 mm).

At 6 weeks posttransplant, she presented with headaches, nausea, and tremors. Her Scr, calcium, and phosphorous were now 2.7, 15.7, and 3.4 mg/dL, respectively. A kidney biopsy showed no rejection but showed extensive tubular damage and calcification (Fig 1). Radiographs showed large periarticular calcifications, with similar findings on a scintigraphic bone scan (Figs 2 and 3). Her 24-hour urinary calcium excretion increased to 1,014 mg. Evaluation of calciotropic hormones demonstrated iPTH 71 pg/mL, suppressed 25-hydroxy vitamin D (20 pg/mL), 1,25-dihydroxy vitamin D (<8 pg/mL), and PTH-related peptide (9 pg/mL). Serum and urine protein electrophoresis was normal, and computed tomography showed no granulomas or lymphadenopathy.

Volume expansion with normal saline at 200 mL/h was initiated in addition to calcitonin (8 U/kg) every 12 hours for 48 hours and pamidronate 60 mg infused over 6 hours. Her prednisone dose was increased to 20 mg daily, and cinacalcet was increased to 90 mg daily. Despite this therapy for 4 days, her calcium only improved marginally to 14.9 mg/dL. Hemodialysis with a low-calcium dialysate (2 mEq/L) was initiated then, given worsening kidney function and hypercalcemia. She received daily 4-hour dialysis sessions; after her fifth and last treatment, her Scr, calcium, and phosphorous improved to 10-11, 1.2, and 3.5 mg/dL, respectively.

**Figure 4:**
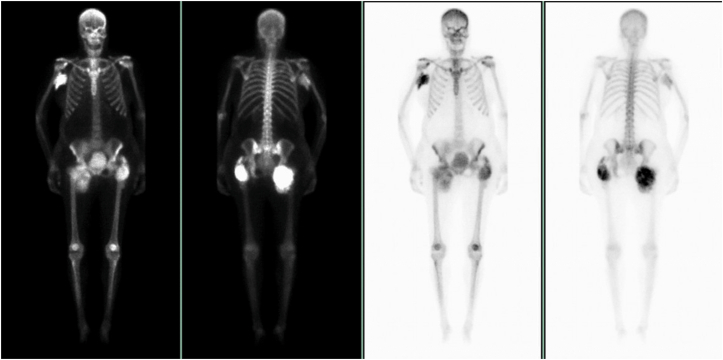
Nuclear medicine whole-body scintigraphy demonstrating bulky periarticular calcifications overlying the posterior right shoulder and bilateral hips.

Despite recovered kidney function, her serum calcium levels remained mildly elevated. This was managed with 8 weeks of saline infusion (2 L) 4 times a week, which eventually normalized her serum calcium level. On the 8-month follow-up, she had normal Scr (1.2 mg/dL), serum calcium (9.2 mg/dL) levels, and 24-hour urinary calcium excretion (246 mg), with near resolution of TC (Fig 4).

## Discussion

TC occurs in around 2% of dialysis patients, characterized by extraskeletal calcified masses in the shoulders, elbows, and hip regions to cause immobility and joint pain.^1^^,^^3^^,^^6^^,^^7^ The accumulation of phosphate owing to reduced renal clearance stimulates the parathyroid gland and causes dysregulation of bone-mineral metabolism, which plays a crucial role in the development of TC. The excess phosphorous binds to the free calcium in the blood, which may be exacerbated by inadequate dialysis, activated vitamin D analogs, and calcium-based phosphate binder use.^1^^,^^3^, ^4^, ^5^

Although TC may typically resolve within a year after transplantation, hypercalcemia is highly prevalent in the first 3 months after transplant and can compromise allograft function, often persisting in 5%-10% of recipients.^8^ Hypercalcemia can cause arteriolar vasoconstriction, which may intensify afferent vasoconstriction associated with calcineurin inhibitors, and also nephrocalcinosis, which can lead to allograft dysfunction.^8^^,^^9^ The patient’s posttransplant phase was marked by an initial improvement of Scr to 1.4 mg/dL. As her hypercalcemia worsened, her allograft function also declined (Scr 1.8 at 2 weeks and 2.7 at 6 weeks). Nephrocalcinosis was confirmed with a biopsy (Fig 1).

Our patient progressively developed severe hypercalcemia shortly after transplantation (12 mg/dL at week 2 and 15.7 at week 6). Although her iPTH levels were not fully suppressed (ie, <20 pg/mL), as would be expected for the severity of hypercalcemia, her hormone level dropped 10-fold from her pretransplant level to 77 pg/mL. The incomplete suppression of iPTH secretion likely reflected autonomous PTH secretion. Adynamic bone disease was considered a potential cause, but chronic elevations in her pretransplant iPTH levels, along with the acute posttransplant changes in her calcium and PTH levels, were inconsistent with a low bone turnover.^10^ Other etiologies, such as occult malignancy, autoimmune and granulomatous disorders, drugs, and dietary factors, were also ruled out.

Similar cases have been reported in patients with TC receiving peritoneal dialysis who were converted to hemodialysis owing to inadequate clearance. In these cases, improvement in phosphate clearance through aggressive hemodialysis has been shown to promote dissolution of TC.^11^^,^^12^ Our patient’s hypercalcemia and acute allograft injury were PTH independent, as evidenced by phosphorus 3.4 mg/dL, elevated urinary calcium, low iPTH, and low vitamin D metabolites.

The management of early posttransplant complications from TC is not well described in the literature. Retrospective studies suggest variable benefits of parathyroidectomy before or after transplantation, with no defined PTH threshold to guide prophylactic surgery.^2^^,^^13^ Complications of parathyroidectomy, which need to be considered, include hungry bone syndrome, postoperative infection, bleeding, and recurrent laryngeal nerve injury.^8^^,^^13^ Despite some reports demonstrating successful treatment using osteoclast inhibitors, our patient’s calcium failed to respond to saline, loop diuresis, calcitonin, and pamidronate for 4 days.^14^ Therefore, on the fourth day, early hemodialysis was initiated because of concerns that her kidney function would not tolerate saline loading. Compared with peritoneal dialysis, low-calcium hemodialysis has been documented to be an effective salvage therapy for hypercalcemia with acute kidney injury and can clear up to one-third of the initial calcium level.^3^^,^^15^ The patient responded well to low-calcium bath hemodialysis (2 mEq/L), with a sustained improvement in her kidney function and calcium level. Because her calcium still remained mildly elevated, saline infusion was initiated to increase glomerular filtration and decrease tubular calcium reabsorption. This therapy normalized her kidney function, serum calcium, and urinary calcium excretion. In addition, her TC was nearly resolved at the 8-month follow-up on imaging.

This case demonstrates how severe pretransplant hyperparathyroidism in the setting of TC can lead to PTH-independent hypercalcemia and allograft injury posttransplant. Early identification of TC before kidney transplantation is crucial to help minimize complications. In retrospect, before her referral for kidney transplant, the severity of her hyperparathyroidism and presence of soft tissue calcifications may have warranted aggressive management of the TC before transplantation. Although parathyroidectomy could have been considered, any potential benefits should be weighed against the risk of serious surgical complications. This case suggests that close monitoring of calcium levels and avoidance of activated vitamin D analogs while dependent on dialysis could also be beneficial. This patient continues to be followed outpatient at the time of this report—18 months after transplantation. She has had complete resolution of her TC, and her bone-mineral metabolism is maintained with a non-calcium-based phosphate inhibitor; she is no longer receiving an activated vitamin D analog. Her calcium levels have been normalized since this hospitalization. Her case highlights the need for individualized bone-mineral risk assessment in kidney transplant candidates with these risk factors.
